# High mobility group box protein-1 (HMGB-1) as a new diagnostic marker in patients with acute appendicitis

**DOI:** 10.1186/1757-7241-19-27

**Published:** 2011-04-20

**Authors:** Yavuz Albayrak, Ayse Albayrak, Muhammet Celik, Ibrahim Gelincik, Ismail Demiryılmaz, Rahsan Yildirim, Bunyami Ozogul

**Affiliations:** 1 Department of General Surgery and Burn Unit, Erzurum Region Education and Research Hospital, Erzurum, Turkey; 2 Department of Infectious Diseases and Clinical Microbiology, Erzurum Region Education and Research Hospital, Erzurum, Turkey; 3 Department of Clinical Biochemistry, Ataturk University, School of Medicine, Erzurum, Turkey; 4 Department of Pathology, Erzurum Region Education and Research Hospital, Erzurum, Turkey; 5 Department of General Surgery, İbni Sina Hospital, Turkey; 6 Department of Internal Medicine, Ataturk University, School of Medicine, Erzurum, Turkey; 7 Department of General Surgery, Erzurum Region Education and Research Hospital, Erzurum, Turkey

## Abstract

**Background**

The aim of this prospective study was therefore to evaluate the diagnostic value of preoperative serum High Mobility Group Box Protein-1 (HMGB-1) levels in patients with Acute Appendicitis (AA) who show normal white blood cell count (WBC) counts.

**Method**

Our study was carried out from October 2010 through November 2010 and included 20 healthy control group participants and 60 patients who presented at the emergency department of Erzurum Training and Research Hospital in Turkey with acute abdominal pain complaints, who were pathologically diagnosed with AA after laparotomy, and who agreed to participate in the study.

**Results**

Of the 60 patients who underwent appendectomies, 36 were male and 24 were female, and of the healthy group, 12 were male and 8 female. The age averages of the patients in Groups 1, 2 and 3 were, respectively, 31.3+15.4, 34.0+16.3 and 31.0+13.1 years. The WBC averages of Groups 1, 2 and 3 were, respectively, 7.41+2.02 (x10^9^/L), 15.71+2.85 (x10^9^/L) and 8.51+1.84 (x10^9^/L). The HMGB-1 levels for Groups 1 (healthy persons), 2 (AA patients with high WBC counts ) and 3 (AA patients with normal WBC counts) were, respectively, 21.71 ± 11.36, 37.28+13.37 and 36.5 ± 17.73 ng/ml. The average HMGB-1 level of the patients with AA was 36.92 ± 15.43 ng/ml while the average HMGB-1 value of the healthy group was 21.71 ± 11.36 ng/ml.

**Conclusion**

The significantly higher levels of HMGB-1 in AA patients compared to healthy persons infer that HMGB-1 might be useful in the diagnosis of AA. Use of HMGB-1, especially in patients with normal WBC counts, will reduce the number of unnecessary explorations.

## Background

Acute appendicitis (AA) is a common abdominal surgical emergency that can affect individuals of all ages, with a lifetime occurrence of approximately 7% [1,2]. AA is commonly diagnosed through a combination of clinical information including symptoms and physical examination findings, traditional biomarkers (e.g., white blood cell count (WBC), mean platelet volume (MPV), absolute neutrophil count (ANC), and C-reactive protein (CRP)) and radiographic imaging (e.g., ultrasound and computed tomography scans) [3-6]. However, preoperative diagnostic difficulties still occur, resulting in a percentage of incorrect diagnoses that can reach up to 20% in the general population, and even up to 40% in women of reproductive age [7-9].

Despite the multiple modern diagnostic tools currently available, diagnosis of AA still depends primarily on patient history and physical examination. The result is that, even with access to laboratory and radiological diagnostic equipment capable of aiding diagnosis of AA, on occasion, patients without actual AA will still undergo unnecessary exploratory surgery. Therefore, the need is great for new, easily applied and inexpensive diagnostic tools that have high diagnostic value for AA and little operator dependence.

The most often used and most practical laboratory test for AA diagnosis is the WBC test. However, WBC counts can sometimes be normal in patients with AA, causing doctors to have difficulties in diagnosing AA. In the present study, we hypothesize that High Mobility Group Box Protein-1 (HMGB-1) could be a good candidate to aid in AA diagnosis. HMGB-1 is viewed as a proinflammatory cytokine due to its active secretion by innate immune cells such as neutrophils, monocytes and macrophages [10,11]. The aim of this prospective study was therefore to evaluate the diagnostic value of preoperative serum HMGB1 levels in patients with AA who show normal WBC counts.

## Methods

### Study populations

The protocol was approved by the Research Ethics Committee of Erzurum Region Education and Research Hospital. Our study was carried out from October 2010 through November 2010 and included 60 patients who presented at the emergency department of Erzurum Training and Research Hospital in Turkey with acute abdominal pain complaints, who were pathologically diagnosed with AA after laparotomy and who agreed to participate in the study. A healthy control group consisted of 20 healthy persons who came to the hospital just for purposes of a check-up in the Infection Diseases Clinics, who had no complaints and who did not conform to the exclusion criteria. Full blood counts were performed on patients who had a history of periumbilical or right lower quadrant pain, who exhibited nausea, vomiting, anorexia, or fever, who showed abdominal examination findings and/or whose condition was indicative of AA based on the general clinical intuition of the physician. Abdominal ultrasound scans were performed on all patients.

### Inclusion and exclusion criteria

All of the patients underwent operations for appendicitis on the basis of the history, physical findings, and relevant clinical data. Postoperatively, the removed appendix was sent for histopathological examination. Cases where the histopathology was not consistent with appendicitis were excluded from the study. The exclusion criteria for entry into the study were heart failure, peripheral vascular disease, haematological disorders, acute or chronic infection, cancer, prior antibiotic therapy, an age < 10 years, pregnancy, hepatic diseases and other known inflammatory conditions. None of the patients had received prior anticoagulant medications, nonsteroidal anti-inflammatory drugs or oral contraceptives.

### Laboratory assays

Blood samples were obtained from the patients upon admission to the hospital. Preoperative complete blood count (CBC) was performed using a Beckman Coulter analyzer (Bayer Healthcare LLC, Diagnostic Division, Tarrytown, New York). The sensitivity, specificity, positive predictive value (PPV) and negative predictive value (NPV) of these tests were calculated. All laboratory analyses were performed in the haematology laboratory of our hospital. Normal values for white blood cells (WBCs) were determined based on published reference ranges from the hospital's haematology laboratory. Preoperative blood samples were collected into Vacutainer tubes (BD, New Jersey), centrifuged at 1300 g for 10 min, and stored at -80°C until the analysis. HMGB1 levels were measured in duplicate using commercially available ELISA kits (HMGB1: Uscn Life Science Inc., Wuhan, China) following the manufacturer's instructions.

### Statistical analyses

A total of 60 patients and 20 healthy persons were included in this study, and were separated into three groups. The first group (Group1) consisted of healthy persons (n = 20), the second group (Group 2) consisted of Acute Appendicitis patients with high WBC counts (n = 32), and the third group (Group 3) consisted of Acute Appendicitis patients with normal WBC counts (n = 28).

The Statistical Package for Social Sciences (SPSS) 16.0 for Windows was used to analyze the data in terms of mean ± standard deviation (SD). Group comparisons were performed using one-way ANOVA of repeated measurements. For post hoc analysis, the Tukey test was applied. AA and control groups were compared using Student *t*-tests. Receiver operating characteristic (ROC) curve analysis was used to identify optimal cut-off values of HMGB-1. Sensitivity, specificity, PPV and NPV were calculated according to standard methods. P values below 0.05 were considered statistically significant.

## Results

### Patient characteristics

Of the 60 patients who underwent appendectomies, 36 were male and 24 were female, and of the healthy group, 12 were male and 8 female. Demographic and clinical characteristics with regard to age, gender distribution, WBC and HMGB-1 levels are presented in Table 1.

**Table 1:** Clinical Characteristics of Acute Appendicitis Patients and Controls

	Group 1 (n = 20)	Group 2 (n = 32)	Group 3 (n = 28)
Sex (M/F)	12/8	20/12	16/12
Age (years) (median, range)	29 (16-77)	27 (17-70)	27 (17-77)
WBC (x10^9^/L)	7.41 ± 2.02	15.71 ± 2.85	8.51 ± 1.84
HMGB-1 (ng/ml)	21.71 ± 11.36	37.28 ± 13.37	36.51 ± 17.74

Group 1: Control group, n = 20; Group 2: Acute Appendicitis patients with high WBC counts; Group 3: Acute Appendicitis patients with normal WBC counts; M: Male; F: Female; WBC: White blood cell count; HMGB-1: High Mobility Group Box Protein-1

### Levels of HMGB-1 in controls and in acute appendicitis patients

**Figure 1:**
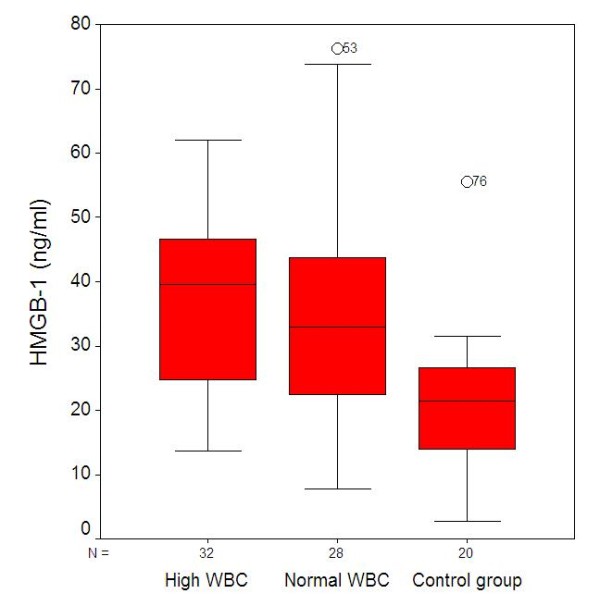
**High Mobility Group Box Protein-1 (HMGB-1) by category of groups**.

The HMGB-1 results for the patient and control groups are shown in figure 1. The average HMGB-1 level of the 60 patients with Acute Appendicitis (AA) was 36.92 ± 15.43 ng/ml, while the average HMGB-1 value of the healthy group was 21.71 ± 11.36 ng/ml. When the groups were compared, the HMGB-1 serum levels were significantly lower (p = 0.001) in the healthy control group. When subgroups were compared, significantly lower serum levels were found for HMGB-1 between the Group 1 healthy controls and Group 2 (p = 0.001) and between the Group 1 controls and Group 3 (p = 0.003). No statistical difference was found between Group 2 and Group 3 (p > 0.05). When all 60 patients with AA were compared by a *t *test with the healthy group, the higher values of HMGB-1 in the AA patients were statistically significant (p < 0.001).

**Figure 2:**
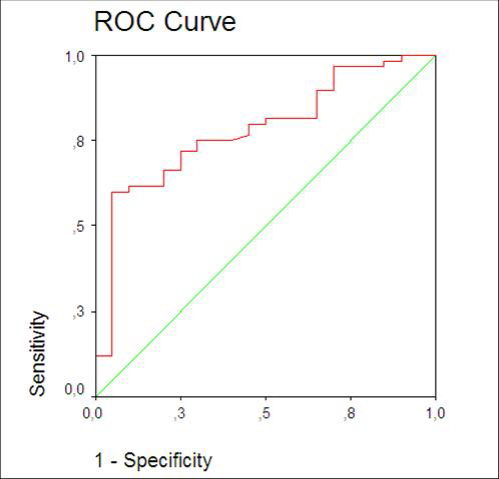
**Receiver operating characteristic (ROC) curve of High Mobility Group Box Protein-1 (HMGB-1)**.

The diagnostic value of HMGB-1 levels was investigated by calculating ROC curves. For the diagnosis of AA, the best cut-off point was at 25 ng/ml. The calculated sensitivity, specificity, positive predictive value and negative predictive value were calculated as 72%, 73%, 88% and 45%, respectively (area under curve = 0.781) (Figure 2).

## Discussion

In this study, we investigated potential variations in the blood levels of HMGB-1 levels that might occur in patients with AA when compared with healthy persons. The overall aim was to determine whether this blood component might have a place in the diagnosis of AA. To our knowledge, no studies reported in the literature have yet evaluated an association between HMGB-1 and AA.

**Figure 3:**
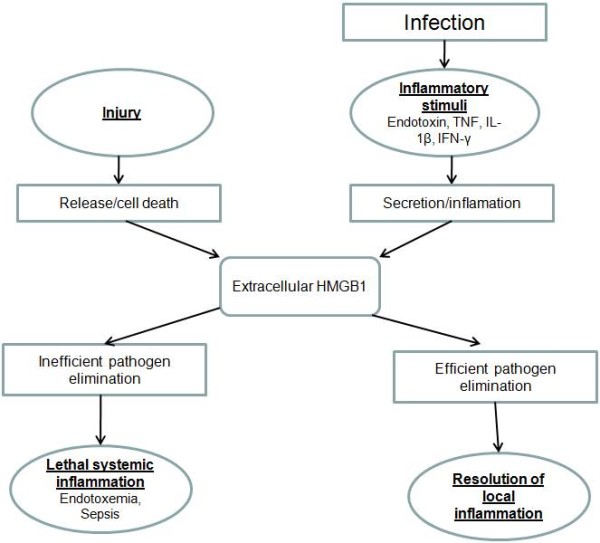
**Schematic summary of the HMGB1 release and action**. HMGB1 can be actively secreted by innate immune cells in response to exogenous microbial products from infection; or passively released from injured or necrotic cells.

HMGB1 (previously designated HMG1 or amphoterin) [12] is not a new protein. It was discovered > 30 years ago as a nuclear DNA-binding protein and was initially named for its characteristic rapid electrophoretic mobility in polyacrylamide gels [13]. HMGB1 is a cytokine that can be released by activated monocytes, macrophages, neutrophils and platelets, and in turn mediates inflammation and enhanced cell motility. Extracellular HMGB1 acts as a late mediator of inflammation. Tracey and collaborators detected HMGB1 in the serum of septic patients [14] and found that application of anti-HMGB1 antibodies reversed established sepsis [15]. In macrophages and neutrophils, HMGB-1 also induces the production of proinflammatory cytokines such as TNF, interleukin (IL)-1a and -1b, IL6 and macrophage inflammatory protein 1 (MIP1) [10,14,16]. During the inflammatory process, HMGB1 migration to organs/tissue sites induces various inflammatory cytokines including TNF-α, IL-1α, IL-1β, IL-1RA, IL-6, IL-8, MIP-1α and MIP-1β, thereby promoting chronic inflammation [17,18]. HMGB1 has been suggested to serve as a pro-inflammatory cytokine [19] and it has many organ-specific biological functions including induction of fever, anorexia, and weight loss, as well as cytokine production in the brain, acute lung injury and production of pro-inflammatory cytokines/mediators in the lungs, promotion of translocation in the gut, induction of arthritis and joint inflammation, modulation of heart rhythm and bactericidal effects [20]. Extracellular HMGB1 translocation during inflammatory responses leads to significantly increased in vivo serum levels in patients with arthritis, sepsis, disseminated intravascular coagulation and other inflammatory disorders [14,21-24]. Increased levels of HMGB-1 have been found following myocardial ischemia, in cerebral ischemia subjects [25] and in chronic kidney disease patients, where this elevation correlates well with inflammatory markers in the synovial fluid as well as with a reduction in glomerular filtrate [26]. When compared to healthy controls, HMGB-1 concentrations in plasma and lung epithelial lining fluid were increased in patients with acute lung injury and acute respiratory distress (ARDS) [27]. In one study, the circulating blood of patients with pneumonia and with pneumonia combined with serious sepsis showed greatly elevated levels of HMGB-1 [28]. In addition, patients with low hepatic fibrosis also showed elevated serum levels of HMGB-1 [29]. In our study, higher HMGB-1 levels were found in AA patients with high WBC counts and with normal WBC counts than in the healthy group. This condition can be explained by the action of HMGB-1, which is a proinflammatory cytokine secreted by the neutrophils, monocytes and macrophages throughout the inflammatory processes involved in AA. Gaini, et al., in the study comparing bacteraemic and non-bacteraemic patients, have determined significantly higher levels of HMGB-1, and have also shown that the increase in HMGB-1 levels correlated with other pro-inflammatory indicators (WBC, CRP and neutrophils). At the conclusion of this study, they have asserted that the high levels of HMGB-1 in bacteraemic patients may be related to the pro-inflammatory role of HMGB-1 [30]. There is a possible mechanism which is the increase of serum HMGB-1 levels in patients with acute appendicitis. HMGB-1, is secreted by stimulated macrophages/monocytes in the late stage of inflammation, may be produced and released by macrophages/monocytes in response to inflammatory mediators. In acute appendicitis, it is conceivable that the release of humoral mediators from the excessive activated macrophages/monocytes may lead to remote organ injury. As the released HMGB-1 can cause the development of inflammation,[31]. release of HMGB1 from activated macrophages/monocytes may participate in tissue inflammation in acute appendicitis [32]. Post infection release and effects of HMGB-1 have been schematized in Figure 3. For these reasons, serum levels of HMGB-1 rise during acute appendicitis. Kosai, et al., have shown that WBC count is correlated with the increase of HMGB-1 in bacterial pneumonia patients co-infected with influenza [33]. In our study, we have also determined a correlation between an increase in HMGB-1 levels and WBC counts in acute appendicitis. However, our study has also shown that in acute appendicitis, an increase in HMGB-1 levels may occur even when there is no increase in WBC numbers. These findings have shown that HMGB-1 serum levels may be used in the diagnosis of acute appendicitis as a non-invasive indicator. HMGB-1 levels are already known to rise during infections or sepsis [14,21,22,28,30-33], but the key result from the current study is that HMGB-1 levels of the AA group with normal WBC counts were significantly higher than those of the healthy group. This shows that HMGB-1 can help to discriminate AA and that it might provide a diagnosis for patients who present with suspected AA, but have normal WBC counts.

## Conclusions

In conclusion, although many auxiliary diagnostic tools are available for diagnosis of AA, this condition is sometimes difficult to diagnose. Misdiagnosis results in many persons undergoing unnecessary exploratory surgeries. Therefore, a need exists for new diagnostic tools that can support a diagnosis of AA. The significantly higher levels of HMGB-1 in AA patients compared to healthy persons infer that HMGB-1 might be useful in the diagnosis of AA. We believe that the use of HMGB-1, especially in patients with normal WBC counts, will reduce the number of unnecessary explorations.

## Abbreviation

HMGB-1: High Mobility Group Box Protein-1; AA: acute appendicitis; WBC: white blood cell count; MPV: mean platelet volume; ANC: absolute neutrophil count; CRP: C-reactive protein; PPV: positive predictive value; NPV: negative predictive value; ROC: Receiver operating characteristic; IL: interleukin; MIP1: macrophage inflammatory protein 1; ARDS: acute respiratory distress syndrome.

## Competing interests

The authors declare that they have no competing interests.

## Authors' contributions

YA, AA and RY are the supervisor of the study., carried out control of and contributed to data extraction and writing of the study. YA and BO operated on the patients. AY and BA contributed to the data extraction. MC performed the biochemical tests. IG performed the histological examination. ID revised the manuscript and painted figure 3. All authors read and approved the final manuscript.
